# One-Step Fabrication of Three-Dimensional Fibrous Collagen-Based Macrostructure with High Water Uptake Capability by Coaxial Electrospinning

**DOI:** 10.3390/nano8100803

**Published:** 2018-10-08

**Authors:** Zahra Bazrafshan, George K. Stylios

**Affiliations:** 1 Organic Chemistry Laboratory, Research Institute for Flexible Materials, Heriot Watt University, Galashiels TD1 3HF, UK; 2 Research Institute for Flexible Materials, Heriot Watt University, Galashiels TD1 3HF, UK; g.stylios@hw.ac.uk

**Keywords:** collagen, conductivity, coaxial electrospinning, bending instability, self-assembly, chain orientation, hydrophilicity

## Abstract

One step fabrication of the three dimension (3D) fibrous structure of Collagen-g-poly(MMA-co-EA)/Nylon6 was investigated by controlling the experimental conditions during coaxial electrospinning. This 3D fibrous structure is the result of interactions of two polymeric systems with a varied capability to be electrostatically polarized under the influence of the external electric field; the solution with the higher conductivity into the inner spinneret and the solution with the lesser conductivity into the outer capillary of the coaxial needle. This set-up was to obtain bimodal fiber fabrication in micro and nanoscale developing a spatial structure; the branches growing off a trunk. The resultant 3D collagen-based fibrous structure has two distinguished configurations: microfibers of 6.9 ± 2.2 µm diameter gap-filled with nanofibers of 216 ± 49 nm diameter. The 3D fibrous structure can be accumulated at an approximate height of 4 cm within 20 min. The mechanism of the 3D fibrous structure and the effect of experimental conditions, the associated hydration degree, water uptake and degradation rate were also investigated. This highly stable 3D fibrous structure has great potential end-uses benefitting from its large surface area and high water uptake which is caused by the high polarity and spatial orientation of collagen-based macrostructure.

## 1. Introduction

In recent years, assemblies of three dimensional (3D) nanostructures are given significant attention due to their desirable effects, such as surface and size properties, which make them suitable for specific applications in many fields [1,2,3]. Among the processing methods for 3D structures, electrospinning is a simple and versatile approach for fabricating ultrafine fibers in a continuous process with diameters fluctuating from micrometers down to tens of nanometers [3,4,5,6].

A typical electrospinning set-up includes a high-voltage source that is connected to the needle of a syringe and a grounded collector for jetted fibers [7]. A positively charged jet emits fibers through a Taylor cone towards a grounded collector [5,8,9,10]. Generally, fibers are randomly deposited on a collector posing a smooth two-dimensional (2D) nonwoven mat since the thickness of collected fibers is not significant [4,11,12,13]. In a similar way, 3D electrospun fibrous structures can also be produced by even better properties than their 2D counterparts, due to their spatial shape, large surface area and pore size. These 3D fibrous structures demonstrate promising superior stability in tissue engineering, energy harvesting, filtration, micro-containers and textiles [4,12,13].

Nevertheless, 3D electrospun fibrous macrostructures are still in their early stages of development and researchers try to fabricate them directly using auxiliary devices (e.g., 3D collectors) [4,6,8,14] or by enhancing the third dimension through increasing time (2 h to 20 h) [1], or by assembly/post-processing of 2D electrospun fibrous structures (e.g., layer-by-layer electrospinning) [11,15,16,17,18] and by self-assembly approaches [3,12,13]. Self-assembly is one of the most interesting approaches to fabricate 3D electrospun fibrous structures. This bottom-up approach can easily fabricate materials into unique structures. For instance, Deitzel et al. reported the formation of a heterogeneous or a 3D structure through the macroscale morphology of electrospun non-woven fibers by increasing the concentration/viscosity of Polyethylene oxide (PEO) with a high molecular weight of 400,000 [19] applying a typical electrospinning with a single spinneret (needle). Thereafter, this method was also tried by different research groups using an increased ionic polarization of the spinning solutions associated with the use of low molecular weight salts (e.g., LiCl, CaCl_2_, M(NO_3_), Fe(NO_3_)_3_), NaCl along with conductivity of PEO [4,8,12,13,14,17,20]. The aforementioned self-assembly fabrication is due to increasing the mass of electrospun fibers which appears in the shape of varied range of fiber diameters (1.2–6.8 µm) [12,13], forming the third spatial dimension for accumulated fibers over time, which can be happened only to polymer solutions with relatively high electrical conductivity and chain entanglements, even though shearing strength and viscosity of electrospinning solutions are likely to be affected by the presence of salts [21].

Collagen is a leading biomaterial with excellent biological and physiochemical properties that can be selected within biomimetic materials [22,23]. Attempts have been made to modify collagen by graft polymerization to benefit from its natural properties while at the same time, add value by introducing monomer(s) to its main chain [24,25]. Thus, far, the main objective of many research groups has been focused on reducing the high hydrophilicity of collagen chains and benefiting in a controllable degradation by using the graft polymerization method [7,26,27,28,29,30]. With the development of nanotechnology coupled with microfabrication techniques, nanostructured materials can closely fulfill the requirement in modern medicine for surgical sponges that can also be developed to deliver drugs, cell and structure [5,31]. Among them, coaxial electrospinning can be a revolutionary development in the field of medicine, science and technology [32].

The goal of this work is to discuss a hybrid of novel macro-structured natural and synthetic polymers; one-step microfabrication and nanofabrication, along with indicating the high water absorption capacity of the resultant fibrous structure for applications requiring super-high-density of water uptake via a superior degree of spatial orientation. In this work, the collagen graft copolymer was tailored with significantly reduced electrical conductivity due to dielectric properties of its side chains. Then, to form a 3D structure, coaxial electrospinning was applied by using two varied polymeric systems with completely different conductivity spun simultaneously; collagen graft copolymer was introduced by the outer capillary (the shell) whereas a solution of Nylon 6 delivering a higher conductivity fed through the inner capillary (the core) of a coaxial needle. The core feeding material can be optionally replaced with any other (bio)polymer having the same properties.

The importance of this work is underpinned by the unique properties of collagen which is featured in this new 3D structure which, to the best of our knowledge, has not been tried before. The produced collagen-based 3D electrospun fibrous structures can be cut into any desired size depending on their biological end use. Hence, this work is particularly important to end uses which need the hydrophilicity of collagen in a 3D structure such as biomedical, filtration superabsorbers and high-performance textiles.

## 2. Experimental Section

### 2.1. Material

Acid soluble Collagen (ASC) from cow skin was provided by Devro Company Inc., Moodiesburn, UK. Methyl methacrylate (MMA, 99%, Alfa Assar, Heysham, UK), Ethyl Acrylate (EA, 99%, Alfa Assar, Heysham, UK) were used as monomers and were passed through a column of 5% sodium hydroxide aqueous solution to remove inhibitor existing in the monomers. Benzoyl peroxide (BPO, 97%, Alfa Assar, Heysham, UK) was used as initiator and recrystallized in Acetone before applying. Distilled water was used as a medium in polymerization process. Acetic acid (AA, 99.7%, Alfa Assar, Heysham, UK), Formic Acid (FA, 97%, Alfa Assar, Heysham, UK), Nylon 6 (N6, 1.086 g·mL^−1^, Sigma Aldrich, Gillingham, UK) and methanol (MeOH, 99.9%, Alfa Assar, Heysham, UK) were applied as received. D&C Red 28 (Rdye, Acid Red 92, Clariant, Muttenz, Switzerland) and Sanolin Tartrazine X90 (Ydye, Acid Yellow 23, Clariant, Muttenz, Switzerland) were used as tint markers to differentiate the core and the shell solutions.

### 2.2. Synthesis of ASC-g-poly(MMA-co-EA)

The procedure of the copolymer synthesis is given in detail in our recent previous work [25,33]. A typical graft polymerization procedure was used for the synthesis of branched ASC-g-poly(MMA-co-EA) (CME). Briefly, Acid soluble collagen (ASC, 11 g) was prepared using collagen in 0.1 M of AA and water to reach pH of 3 ± 1. The mixture was incubated for 5 h at 45 °C in a 250-mL triple necked round bottom flask and a stirrer bar was added. Subsequently, free radical polymerization was used to synthesize the graft copolymers of MMA-co-EA onto ASC in water. Once the desired temperature of 80 °C was achieved, the gentle addition of BPO (13.62 × 10^−4^ M) dissolved in Acetone, as the initiator, was added to the reaction vessel within 10 min. MM-co-EA (329.65 × 10^−4^ M, 10% EA content) was then introduced via a syringe in 30 min. The temperature and the reaction time after adding the initiator and the monomers were fixed at 80 °C and 60 min. The reaction mixture was then added to rapidly stirring cool MeOH for complete precipitation. Then the solution was filtered and dried in a vacuum oven in 25 °C until a constant weight was obtained. To remove ungrafted ASC, the grafted copolymer was treated in boiling water by changing the water three times in 2 h, and then was refluxed with excess acetone and stirred for 2 h to remove poly(MMA-co-EA). The resultant collagen graft copolymer (CME) was dried in a vacuum oven at room temperature until constant weight was achieved.

### 2.3. Preparation of Spinning Solutions and Coaxial Electrospinning

FA was used as the solvent of both solutions of core and shell components. The core solution consisted of N6 and Rdye in FA by stirring for 4 h at room temperature. The shell solution made up of CME and Ydye dissolved in FA within 5 h at room temperature. The core solution was loaded into a 10-mL syringe and the shell solution into a 5-mL syringe. Detailed sample description and the solution properties and the electrospinning process conditions are shown in Table 1 and Table 2.

To follow the electrospinning process, two distinguished dye markers in shade, with no overlay on color spectrum were used (Red and Yellow) in small amounts to minimize any chemical effect on associated electrospun fibers. The positively charged coaxial needle (gauge 22 & 26) was used as spinneret. The Spreybase^®^ electrospinning system was applied consisting of a typical electrospinning process setup which included a high voltage source that was connected to the coaxial needle linked to a syringe by a polytetrafluoroethylene (PTFE) tubing, and a grounded collector. To study the size and morphology of the electrospun fibers, Scanning Electron Microscopy (SEM, Hitachi S-4300, Hitachi Ltd., Tokyo, Japan) and the mean fiber diameter and uniformity of the fibers were estimated statistically by using ImageJ software from SEM micrographs (*n* = 80, 1:1 nano-:micro-fiber). The electrospun fibers were coated with a gold thin film before SEM imaging to ensure higher conductivity. To determine fiber alignment, Fast Fourier transform (FFT) was performed using ImageJ on representative sample images. The reverse FFT data FFT images were then analyzed in directionality profile, wherein the radial intensity was summed and plotted with regards to the angle of orientation. A digital camera was used within the spinning system to observe and record the spinning process and the resultant 3D fibrous structure. The conductivity value of the sample solutions was determined using a conductivity meter (OAKTON, RS232 CON 110 series, OAKTON Instruments, Vernon Hills, IL, USA). Surface tension studies of the solutions were carried out by using a tensiometer (KRŰSS GmbH, Hamburg, Germany). Viscosities of polymer solutions in FA were determined using a Brookfield DV-II+Pro Viscometer (Brookfield Co., Stoughton, MA, USA) at (20 ± 0.2) °C. To study the water uptake, absorption, and degradation, the collagen graft copolymer/N6 fibrous membranes were first punched into small round pieces with the approximate diameter of 2 cm with a total mass of ∼50 mg. Hydration degrees (water absorption) were studied by weighing the fiber samples (*n* = 3) before and after immersion in distilled water for 24 h. Excess water was removed from the samples by gently blotting with filter paper prior to each weighing while water uptake was measured as removed from the water. The degradation evaluations were achieved as defined by Zhu et al. [34]; the round samples (*n* = 3) were immersed in 70 mL of 154 mM phosphate buffered saline (PBS, pH 4), containing 0.02% sodium azide as a bacteriostatic agent. The suspension was maintained in a controlled temperature of 37 °C. At predetermined time intervals, the reduced buffer was added back with fresh PBS for continuing incubation.

## 3. Results and Discussion

### 3.1. Synthesis of ASC-g-poly(MMA-co-EA)

Details of the synthesis of the collagen graft copolymers have been reported in our recent previous work in which grafting polymerization of MMA-co-EA was used to modify the surface of Acid Soluble Collagen (ASC). We realized that the structurally branched hydrophilic collagen graft copolymer (CME) significantly influenced the initial viscosity of solutions and demonstrated a significant reduction in the conductivity of the branched copolymer, where the side chain copolymer with dielectric properties covalently bonded onto the collagen (supplementary data, Figure 1). The increased viscosity of the solutions in low concentrations confirms the availability of high chain entanglements that resulted from intermolecular interactions of the structurally branched collagen graft copolymer [7]. In this study, we used CME in a solution with specifications that have been shown in Table 1.

### 3.2. Factors to Control CME/N6 3D Fiber Structure

To understand the response of applied solutions to coaxial electrospinning conditions, the electrospinning of each solution was tailored, and Table 2 reveals the associated controlled parameters and observations of each solution that electrospun individually. In electrospinning, the polymer jet from CME solution was initiated to be spun at the applied voltage of about 5 kV and with increasing the intensity of the electrical field, no apparent alteration in fiber spinning behavior was noticed. The spun fibers were like a visible aligned thread that linked the collector to the spinneret. By contrast, the fiber spinning from the N6 solution when electrospun individually demonstrated a different behavior. The polymer jet was initiated at much higher voltage intensity at 14 kV and above. The electrospun fibers were then randomly deposited on the surface of the collector in a 2D shape as none-woven mats. This suggests that in electrospinning, the CME solution has a sufficiently low amount of free surface charge, so that the bending instability is prevented from initiating, while the N6 solution has a sufficiently high free charge, so that its charge relaxation time is too short to form a stable jet.

**Figure 1:**
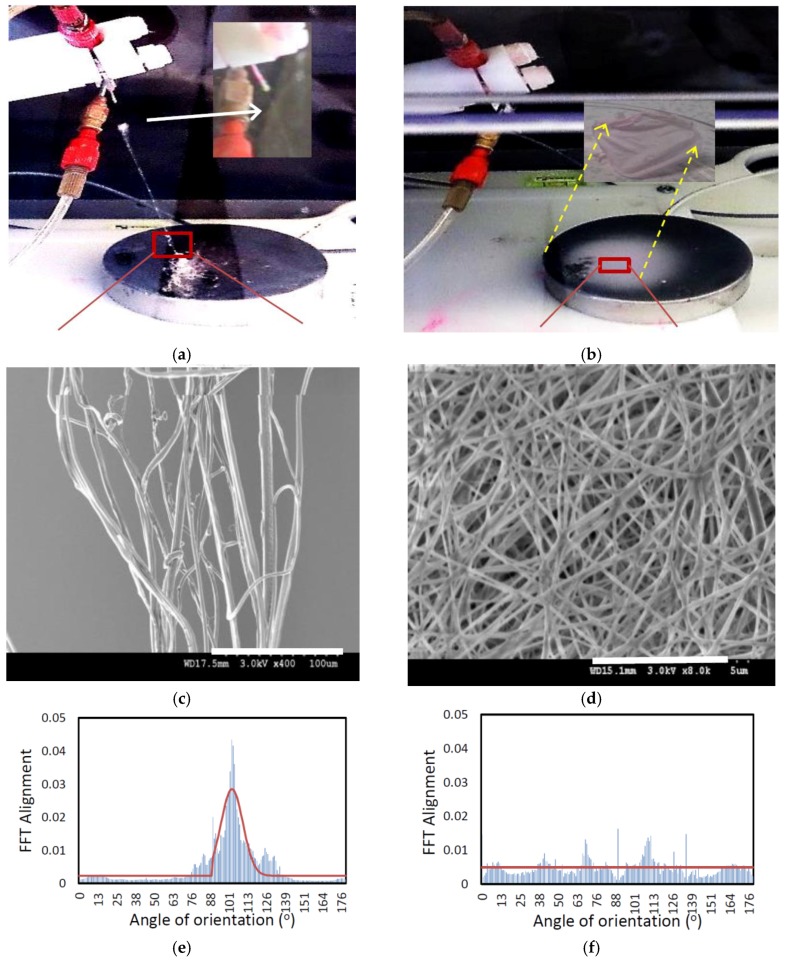
Fiber fabrication and characterizations that were formed in two applied voltage of 9 kV (**a**,**c**,**e**) and 18 kV (**b**,**d**,**f**); (**a**) a bunch of fibers aligned between the collector and the coaxial needle from the shell solution in light yellow color. (**b**) A random fiber orientation from the core solution in pink color. The morphology of the electrospun fibers applying (**c**) 9-kV voltage, (**d**) 18-kV voltage. (**e**,**f**) The directionality histogram reporting 2D-FFT alignment based on peak shape and the relative principle axis of orientation for the fibers that extracted from Image J raw output of FFT analysis of fibers corresponding to SEM images of **c** and **d**.

When the solutions are spun together in a coaxial configuration, only the yellowish solution of CME with the lower conductivity in the shell was spun by applying voltage less than 10 kV. The aligned visible fiber formed towards the grounded collector, as seen in Figure 1a,c,e, while at the same time, the solution in the core with the higher conductivity shown in red color was dropping. The fibers on the collector acted like CME solution as when it was electrospun individually. They started to twist together while growing towards the spinneret and fabricated a yarn-like connected to the tip of the needle in a few seconds. In other words, the electrostatically initiated shell solution was stabilized, leading to a stable, linear jet without a bending instability while the core solution could be partially spun, being assisted by the shell solution and it could be also partially discharged from fiber formation. The process was then interrupted for a while until the fibrous bridge between collector and spinneret was disconnected. In voltages above 18 kV, a pinkish 2D nonwoven mat is gradually fabricated, Figure 1b,d,f. Details of the process parameters and the associated observations of the coaxial electrospinning process are also noted in Table 2.

This comparison confirms that in coaxial electrospinning, the solutions can be charged independently, even though they are employed in the same process conditions and that the prominent driving liquid is the one with the larger electrical conductivity. This is also in agreement with the theoretically proven phenomenon that fiber orientation becomes random as the conductivity of the solution increases, indicating a higher surface charge density to exceed a critical threshold for initiating the bending instability [35,36,37]. More specifically, the core and the shell solutions followed comparatively the same behavior as spun individually; the shell polymer with a low forward jet moving velocity fabricates a relatively high chain alignment toward the spinneret and the core polymer with a highly driven bending instability fabricates a random fiber orientation, Figure 1e,f. This bimodal behavior of the electrospun materials has been experimentally observed in the random deposition style of the core solution over the collector and fiber alignment between two electrodes (the tip of the needle and the collector), Figure 1.

**Figure 2:**
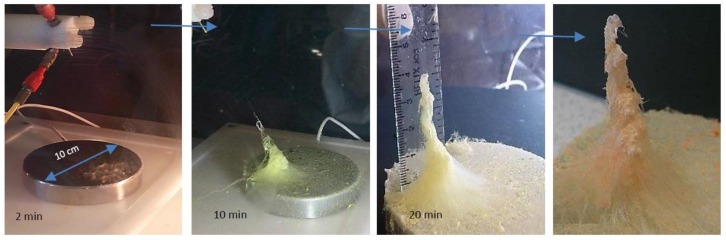
The process of fabrication of the 3D fiber stacks (due to color retention failure using various instruments, the real shade of fibers is slightly changed in some photos).

**Figure 3:**
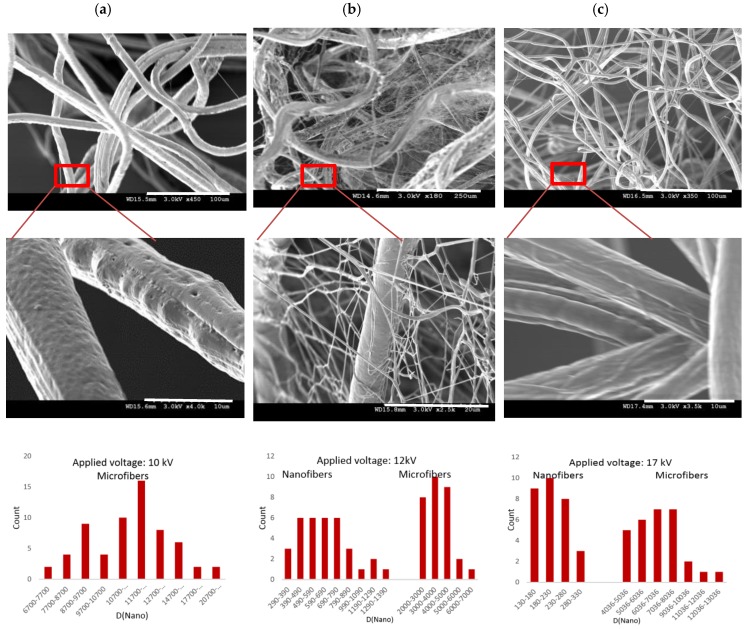
SEM micrographs with varied magnificent for more clarity and associated fiber diameter scattering of micro and nanofibers fabricated in one step coaxial electrospinning with increasing the intensity of the electric field from left to right; (**a**) Microfibers with non-uniform surface (10 kV); (**b**) Micro and nano fibrous network (12 kV); (**c**) Micro and nanofibers that mostly stuck to each other (17 kV).

The typical feature that is associated with coaxial electrospinning methods, is their flexibility to be used for a custom-built fiber formation and structures. Hence, we found that the 12-kV applied voltage is the critical threshold in which two applied solutions collaborate in fiber formation as noticed by the distinct color of the dye markers (Figure 2) and their bimodal fiber characterizations has been shown in Figure 3b. In this case, the onset of the simultaneous bimodal fiber formation is a function of the intensity of electrical field, which is strongly coupled with the electrical properties of the components being delivered through the coaxial needle.

Generally, the coaxial electrospinning process has shown that to fabricate a single fiber from two components, the conductivity of the core components is not essential while the shell solution is required to be of a higher conductivity [38]. In fact, the higher conductivity of core solution is more likely to cause the breakup of the core solution due to the availability of a high surface charge density acting as a strong electrostatically pulling force to form a polymer jet [39].

This signifies that the stability of an electrostatically driven polymer jet is dependent on the interactions of rheological properties and surface charge; when the induced surface charge can have a long enough lifetime to interact with the external electric field, it can generate a tangential electric force that balances the natural tendency of the polymer jet to undergo breakup via the Rayleigh varicose instability [40,41]. When the conductivity is such that surface charge exceeds a critical threshold, then the Rayleigh instability can be reinforced, leading to break up and moving with a higher velocity.

This theory has been already established for single fiber fabrication from two components through coaxial electrospinning [9,35]; however, we have applied it in a way that obtain the bimodal fiber formation instead, which can form interfacial interactions in three dimensions. In practice, the experimental observations from the fiber morphology obtained from the 12-kV applied voltage (Figure 3b), confirm our theoretical predictions of fabricating bimodal fibers simultaneously. The highly conductive solution in the core was employed to benefit from its high moving jet velocity due to high surface charge density under an electrical field to fabricate a spatial design rather than a single fiber fabrication from two components that is commonly associated with coaxial electrospinning.

### 3.3. The Growth Process and Morphology of the 3D Nanofibrous Structure

Coaxial electrospinning at the applied voltage of 12 kV is started with some visible aligned yellowish fibers that are formed on the collector. Then numerous twisting microfibers form vertically all over the collector in varied lengths of about 2–5 cm, rapidly converting to elliptical and coiled accumulations under the area of the spinneret, where the fibrous stack is to be fabricated. This formation persisted in 2 min approximately, Figure 2.

Then, the development of coiling and rolling fibers accumulating over the stack is continued while, simultaneously, the inner diameter of the stack is reduced, as shown in Figure 2 (10 min). A cone-like stack is appeared condensing and growing over time. After almost 20 min, the chaotic formation become dominant again. The process is stopped after 20 min when the approximately 4-cm height of the 3D stack is achieved. As shown in Figure 2 and Figure 3b, the height of the 3D stack is large and dense between the tip of the spinneret and the collector with applied voltages of +12 KV.

Figure 3 also shows the SEM micrographs and the associated fiber diameter scattering of the spun fibers at 3 applied voltage conditions; 10, 12, 17 kV. According to Figure 3b, bimodal fiber formation morphologies are specifically notable at 12 kV; the assembly of micron size fibers (6.9 ± 2.2 µm), gap-filled with a web-like structure of nanofibers (216 ± 49 nm) constructed a 3D fibrous structure. Hence, by controlling the experimental conditions, stated in Table 2, one step quick fabrication of irregular 3D nanofibrous stacks of collagen-based material with the collaboration of the web-like structure of N6 nanofiber is obtained.

**Figure 4:**
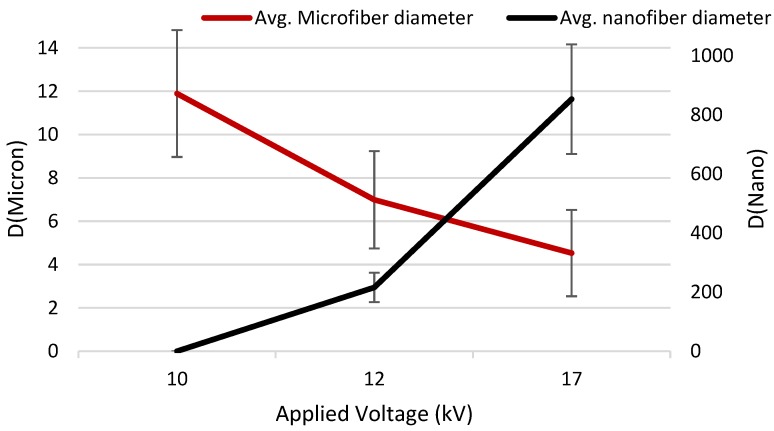
The effect of applied voltage on mean fiber diameter and uniformity of the fibers (standard deviation value), whereas the microfibers were formed from collagen graft copolymer and nanofibers were processed from Nylon 6 (*p* < 0.005).

In addition, Figure 4 demonstrates that the average microfiber diameter that is associated with the shell solution tends to decrease sharply by increasing the applied voltage. By contrast, although no nanofiber is observed in the case of 10 kV applied voltage; it is estimated that the nanofiber diameter is more likely to increase by increasing the electric field. Therefore, more uniformity is observed in the high voltage of 17 kV whereas the volume of the web-like structure is significantly decreased (Figure 3) and the average fiber diameter tends to reach the same point on the graph. This suggests that by adjusting the intensity of the electrical field various fiber formations in design with different contents can be fabricated by coaxial electrospinning.

### 3.4. Self-Assembly Mechanism of 3D Fiber Stack

Collagen has partially cationic behavior [42,43] but the dielectric properties of Poly(MMA-co-EA) covalently bonded on the surface of ASC as side chains, are more likely to overcome vigorously due to low electron mobile phase and electrostatic polarization under the influence of the external electric field that is considered here by means of the lowered conductivity of the CME solution in contrast with the other solution consisting of N6 polymer [7,33].

We applied coaxial electrospinning to consider the effect of the orientation polarization of the polymers in the core and the shell with a varied capacity of built-in dipoles that are independent of each other, i.e., they can move and align individually [44]. By means of the external electrical field, we expected a significantly varied chain orientation due to different polarization dimensions that lead the spun fibers to the minimum of the dipole energy (the grounded collector). Greater polarization orientation can be occurred by the larger factor of the induced surface charge density under the influence of electric field [45,46].

**Figure 5:**
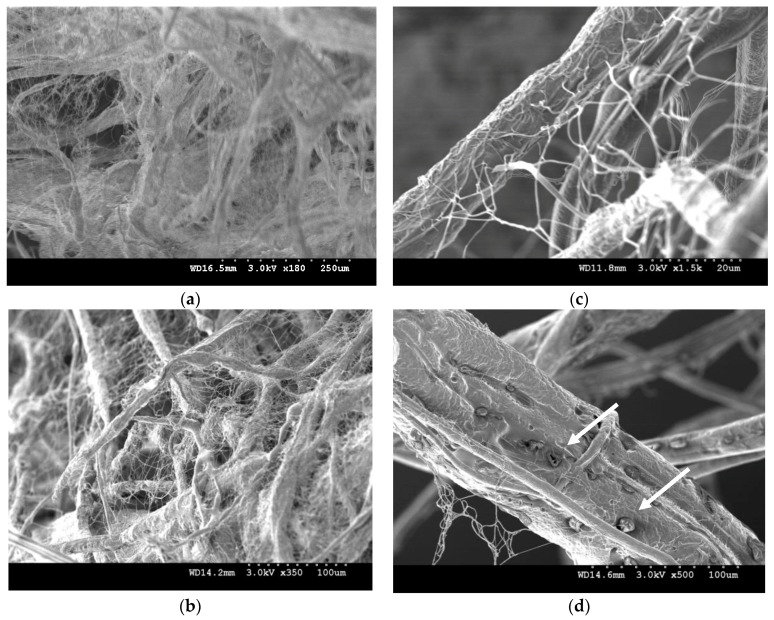
The morphology of bimodal fibers to predict the self-assembly mechanism of 3D fiber stack fabricated at 12 kV-applied voltage; spatial orientation of the 3D macrostructure: (**a**) ×180 (**b**) ×350 (**c**) ×1500 (**d**) Nanofibers from the core solution are likely to pass through weak spots of polymer jet from the shell solution while the solvent evaporates away; this can be shown by the holes (pointed by the white arrows) that remained on the surface of the microfibers of collagen graft copolymer.

For each spinnable polymer solution, the solution conductivity must be in a range that often referred to as a “Leaky Dielectric” which develop electrohydrodynamics referring the deformation/motion of droplets by an electric field [40]. The leaky dielectrics and their responses to electric fields are discussed in detail in the references by Hohman et al. [36,47], as well as Schnitzer et al. [44] and in an earlier work by Saville [40]. With the assumption that the varied capacity of built-in dipoles in leaky dielectrics appears in the shape of a different dimension of polarization; hence, it can be developed that the surface charge density term can be formed depending upon the scaling of relevant orientation and dimension of build in dipoles. This can emerge a jump in voltage across the liquid-liquid interface between the two leaky dielectric media being placed in the same intensity of the electric fields; the varied chain orientation arises as a natural consequence of the interfacial boundary conditions for the dipoles, and that can be significantly detected under certain electrospinning conditions. Our observations also reveal the structure of the varied chain orientation at the liquid-liquid interface (Figure 5), which shows how interfacial chain orientation may arise under strong imposed electric fields. This also submits that the liquid polymer jets can be drifted apart under an imposed electric field depending on the varied velocity of which is appeared obviously to leading order of the solution in the core.

More specifically, there is a possible explanation for the formation of the 3D fiber structure. One particular moment in time, the highly charged polymer jet of N6 solution in the core completely fills the core space and also the shell polymer (CME) is influenced by the impulsion of the dipoles oriented of N6 due to owning the effect of the greater polarization orientation within the electric field. By increasing the intensity of the electric field, N6 is initiated to make channels to pass through the shell polymer and move independently. Therefore, this breakup is more likely to occur at weak spots of CME when a certain voltage is reached. This orientation can be confirmed by the uneven and irregular surface of the microfibers obtained from the 10 kV voltage, Figure 3a. This development is followed by obvious pores on the surface of microfibers, as seen in Figure 5d when the 12-kV voltage is applied. By further increasing the intensity of the electric field, the highly initiated N6 chains influence the shell polymer and drive it more appropriately in a leading order to deposit on the minimum of the dipole energy. This is evidenced by more uniform fibers at 17 kV applied voltage.

By contrast, the case of the low positively chargeable solution is used in the shell to obtain polymer jets with low moving velocity and significantly reduced electrically driven bending instability, and finally a relatively stable and linear movement which is achieved due to the dielectric properties of entangled grown side chains. The resulting continuous fibers in the micron scale act as a trunk that allows the highly electrostatically oriented nanofibers from the core solution to pass through and randomly grow as branches. It is also possible that each nanofiber tends to be absorbed by either its own trunk (microfiber) or any less electrostatically oriented microfiber in its neighborhood, which makes a 3D stack, bulky and gap-filled with nanofibers. This behavior plays the key role for the 3D configuration in which, according to SEM micrographs (Figure 5) occurs in field strength of 12 kV.

As the solvent evaporates away and then the bimodal electrospun fibers probably form a weak secondary electrostatic field together while being led separately to the minimum of the dipole energy (the collector). This is due to the electrostatic forces, so that the fibers are more likely to attract fibers of different origination and repel fibers with the same origination. This causes accumulations on top of each other and makes the fiber deposition appearing as a 3D spatially constructed assembly, Figure 5a. To understand this phenomenon, a negatively charged rod placed close to the edge of the fibers obtained from each solution. It was observed that the negatively charged rod repelled a bundle of aligned fibers from CME and attracted the nano 2D fibers from N6. It is possible that the tip of the cone-like stack reacts as a new collector, where the freshly formed fibers deposit on the top of the stack and hence making it to grow faster in a short time. However, it is likely over time, the severity of the mentioned secondary electrostatic field deteriorates by approaching and growing the stack towards the spinneret as the main source of high DC voltage that is appeared by condensing and reducing the inner diameter of developing cone-like stack, Figure 2. It means that there are some interactions between the residual surface charge on the deposited fibers and the electric field that acts to stabilize the shape of the stack into a 3D shape.

Even though the precise insight of the self-assembly mechanism of 3D fiber stack seems to be as a result of a series of interacting factors, we attempted to keep the process and solutions fixed during coaxial electrospinning process to study the effect of the varied conductivity of both components in the core and in the shell. Our results and observations supported the above-mentioned theory and helped us to understand this fast-self-assembly fabrication of the 3D fibrous structure from varied polymers on a stationary collector. However, this is expected to be changed significantly if the collection target was changed. For example a rotating drum or conveyer belt that can be used for custom-built electrostatically reinforcement [33] of macrostructure fibers, a combination of isotropic and anisotropic fibrous structures, since the control provided by elimination of the bending instability delivers an opportunity for better prediction of the jet movement and deposition.

### 3.5. Wettability, Water Absorption and Degradation Properties of the 3D Fibrous Structure

**Figure 6:**
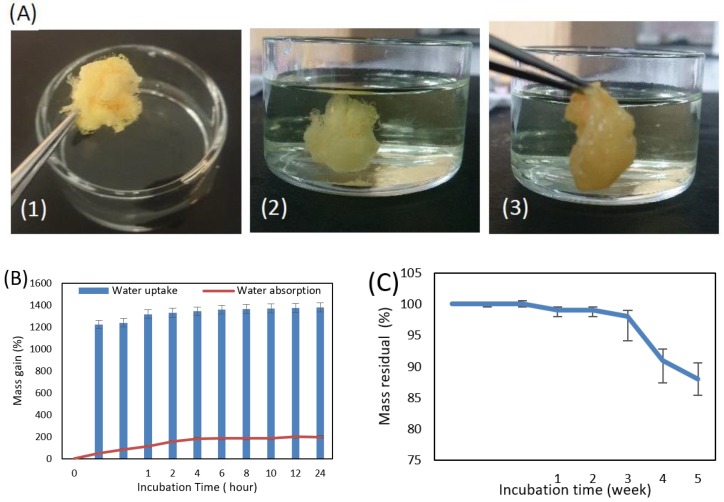
Water absorption behavior (hydration degree) of the 3D fibrous structure of collagen-g-poly(MMA-co-EA)/N6 formed by coaxial electrospinning at 12 kV applied voltage: (**A**) The feature of a sample before immersion in water (dry) (1), in the water after 24 h (2) and out of the water after 24 h (wet) (3). (**B**) Water absorption (hydration degree) and water uptake of samples (*n* = 5, *p* < 0.01) in percentage within 24 h. (**C**) Mass residual percentage of the samples (*n* = 5, *p* < 0.005) in PBS vs. incubation time (week).

To further understand the hydrophilicity and water uptake of the 3D fibrous structure, we studied its water absorption, and degradation properties, taking into account the critical role that water plays in (bio)materials. Figure 6 shows the water absorption behavior (hydration degree) of the 3D fibrous structure of CME/N6 formed by coaxial electrospinning at 12 kV applied voltage; the sample before immersion in water (dry), in the water after 24 h and out of the water after 24 h (wet).

Water absorption (hydration degree) and water uptake of samples in percentage were measured within 24 h. While no significant change was noticed in the water uptake over time, the water absorption was steadily increased after 4 h. The instant water uptake at t(0) (water trapped in fibrous structure) represents the high wettability of the 3D fibrous structure in which the corresponding mass gain is about 1200 g·g^−1^%. The initial weight of the samples achieved about 100 g·g^−1^% growth due to highly hydrophilicity of the fibrous stack, Figure 6B. Water absorption predicts the hydration/swelling degree of the 3D fibrous structure as a function of time. These results indicate the high polarity of the N6 that causes water to easily penetrate through the fibrous stack along with their inherent surface behavior of hydrophilic collagen graft copolymer. From incubation in the PBS buffer for 5 weeks (Figure 6C), it can be observed that the mass residual of the fibrous stack was found to have a significant decrease to 95% during the fourth week. This result suggests that the lifetime of the 3D fibrous stack is predicted to be at least 3–4 weeks without any specific degradation in high humidity situations. The fibrous structure of the samples did not lose their morphology by the end of this test.

## 4. Conclusions

In this work, we have introduced a novel technique for fabricating a 3D nanofibrous collagen-based material. By controlling the experimental conditions, one-step fabrication of irregular 3D fibrous structure filled with web-like nanofibers was obtained in 20 min. via coaxial electrospinning. A key finding in our investigations is the effect of surface charge density, provided by the varied built-in dipoles of the core and the shell solutions that can appear to form a trunk of grown branches, which results in a spatial fiber orientation. In addition, since there can be some interactions between the residual surface charge on the deposited fibers and the electric field, the bimodal fibers in micro and nanoscale are accumulated on top of each other and enable the fiber deposition to form the shape of the fiber stack into a cone-like 3D shape. The importance of this work is in the unique properties of the super-high-density of this material capable of dramatic water uptake induced by a high degree of spatial orientation of the collagen-based material that has the potential of allowing the fibers to transfer liquid, delivering drugs and cells by rapid fabrication and ease of further shaping into a desirable product by cutting.

## Figures and Tables

**Table 1:** Properties of the electrospinning solutions.

Solutions	Viscosity (Pa·s)	Surface Tension (mN/m^2^)	Conductivity (ms/cm)
(CME, 10 wt/v% + Ydye, 0.1 wt% ) in FA	5.87	33.3	1453
(N6, 25 wt/v% + Rdye, 0.1 wt%) in FA	5.97	37.8	4416

**Table 2:** The controlled process parameters and the associated observation.

**Solution ^(a)^**	**Distance (cm)**	**Voltage (kV)**	**Flow Rate (mL/h)**	**T (°C)**	**RH (%)**	**Observation**		
(CME, 10 wt/v% + Ydye, 0.1 wt%) in FA	10	5–10	0.5	22 ± 2	35 ± 5	One end of continuous electrospun fiber touches the collector. A bunch of aligned fibers was formed in yellow color.		
	10	12–20	1.5	22 ± 2	35 ± 5			
(N6, 25 wt/v% + Rdye, 0.1 wt%) in FA	10	5–12	0.5–1	22 ± 2	35 ± 5	Dropping; no Taylor cone angle, no electrospun fiber was observed.		
	10	14–20	0.5–1	22 ± 2	35 ± 5	2D nano mat in a shade of light pink is achieved.		
**Core Solution ^(b)^**	**Shell Solution**	**Distance (cm)**	**Voltage (kV)**	**Flow Rate (mL/h)**		**T (°C)**	**RH (%)**	**Comments (Coaxial CME/N6)**
				**Core**	**Shell**			
(N6, 25 wt/v% + Rdye, 0.1 wt%) in FA	(CME,10 wt/v% + Ydye, 0.1 wt%) in FA	10	9.5–10	0.5	0.5	22 ± 2	35 ± 5	Occasionally droplets of N6 were observed.
		10	17–17.5	0.5	0.5	22 ± 2	35 ± 5	Chaotic CME polymer jet; whipping in the air.
		10	12–12.5	0.5	0.5	22 ± 2	35 ± 5	No droplet was observed.

^(a)^ The process parameters when they were electrospun solely. ^(b)^ The coaxial electrospinning process conditions.

## Supplementary Materials

Supplementary File 1
