# Supplementary figures and images for: One-Step Fabrication of Three-Dimensional Fibrous Collagen-Based Macrostructure with High Water Uptake Capability by Coaxial Electrospinning
Source: Nanomaterials (Basel). 2018 Oct 8;8(10):803. doi: 10.3390/nano8100803 (PMC6215112; doi:10.3390/nano8100803)

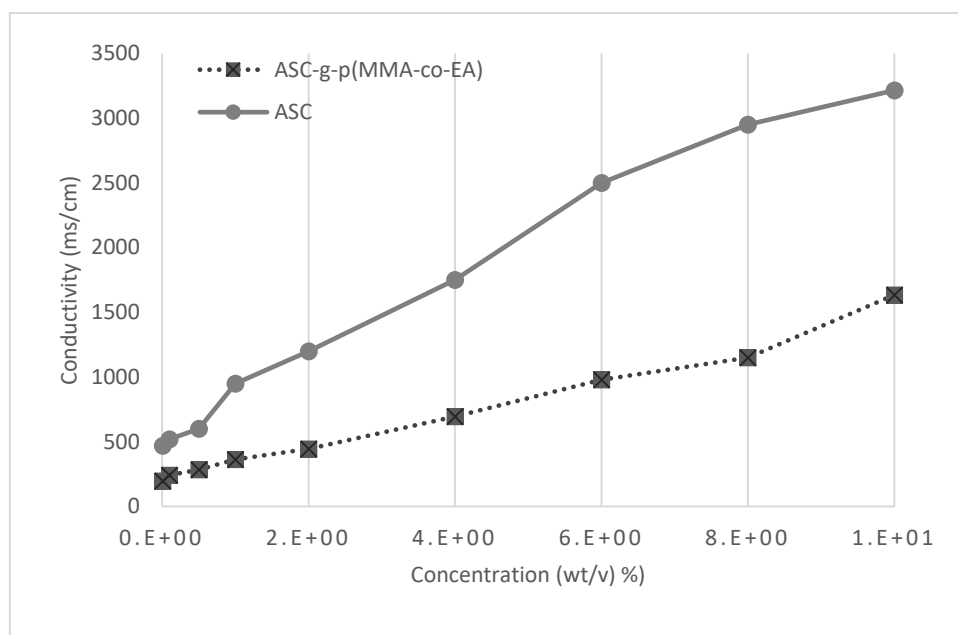

Figure 1 Effect of the presence of Side chains on conductivity of ASC-g-P(MMA-co-EA).

Supplement: Supplementary File 1 [file nanomaterials-08-00803-s001.pdf]
